# Supplementary material for: Mobile apps for detecting falsified and substandard drugs: A systematic review
Source: PLoS One. 2021 Feb 4;16(2):e0246061. doi: 10.1371/journal.pone.0246061 (PMC7861418; doi:10.1371/journal.pone.0246061)
Supplement: S2 File — (DOCX) [file pone.0246061.s002.docx]

## Supporting Information

**S2 Search strategy** (01/07/2020)

**PubMed**

| **#** | **Query** |
| --- | --- |
| [#46](https://www.ncbi.nlm.nih.gov/pubmed) | (#28 AND #45) |
| [#45](https://www.ncbi.nlm.nih.gov/pubmed) | (#29 OR #30 OR #31 OR #32 OR #33 OR #34 OR #35 OR #36 OR #37 OR #38 OR #39 OR #40 OR #41 OR #42 OR #43 OR #44) |
| [#44](https://www.ncbi.nlm.nih.gov/pubmed) | PillsBot[tiab] |
| [#43](https://www.ncbi.nlm.nih.gov/pubmed) | Pillidentif*[tiab] |
| [#42](https://www.ncbi.nlm.nih.gov/pubmed) | Pill Finder[tiab] |
| [#41](https://www.ncbi.nlm.nih.gov/pubmed) | Pill ID[tiab] |
| [#40](https://www.ncbi.nlm.nih.gov/pubmed) | Pill Identif*[tiab] |
| [#39](https://www.ncbi.nlm.nih.gov/pubmed) | Medication Authentic*[tiab] |
| [#38](https://www.ncbi.nlm.nih.gov/pubmed) | Drug Authentic*[tiab] |
| [#37](https://www.ncbi.nlm.nih.gov/pubmed) | Pill Authentic*[tiab] |
| [#36](https://www.ncbi.nlm.nih.gov/pubmed) | Substandard[tiab] |
| [#35](https://www.ncbi.nlm.nih.gov/pubmed) | Fraudulent*[tiab] |
| [#34](https://www.ncbi.nlm.nih.gov/pubmed) | Imitation*[tiab] |
| [#33](https://www.ncbi.nlm.nih.gov/pubmed) | Adulterated[tiab] |
| [#32](https://www.ncbi.nlm.nih.gov/pubmed) | Fake[tiab] |
| [#31](https://www.ncbi.nlm.nih.gov/pubmed) | Falsified[tiab] |
| [#30](https://www.ncbi.nlm.nih.gov/pubmed) | Counterfeit[tiab] |
| [#29](https://www.ncbi.nlm.nih.gov/pubmed) | Counterfeit Drugs[Mesh] |
| [#28](https://www.ncbi.nlm.nih.gov/pubmed) | (#1 OR #2 OR #3 OR #4 OR #5 OR #6 OR #7 OR #8 OR #9 OR #10 OR #11 OR #12 OR #13 OR #14 OR #15 OR #16 OR #17 OR #18 OR #19 OR #20 OR #21 OR #22 OR #23 OR #24 OR #25 OR #26 OR #27) |
| [#27](https://www.ncbi.nlm.nih.gov/pubmed) | eHealth[tiab] |
| [#26](https://www.ncbi.nlm.nih.gov/pubmed) | Telehealth[tiab] |
| [#25](https://www.ncbi.nlm.nih.gov/pubmed) | mHealth[tiab] |
| [#24](https://www.ncbi.nlm.nih.gov/pubmed) | Mobile Health[tiab] |
| [#23](https://www.ncbi.nlm.nih.gov/pubmed) | iPhone[tiab] |
| [#22](https://www.ncbi.nlm.nih.gov/pubmed) | iPad[tiab] |
| [#21](https://www.ncbi.nlm.nih.gov/pubmed) | Handheld Device[tiab] |
| [#20](https://www.ncbi.nlm.nih.gov/pubmed) | Computers, Handheld[Mesh] |
| [#19](https://www.ncbi.nlm.nih.gov/pubmed) | QR[tiab] |
| [#18](https://www.ncbi.nlm.nih.gov/pubmed) | Smartphone*[tiab] |
| [#17](https://www.ncbi.nlm.nih.gov/pubmed) | Cellular Telephon*[tiab] |
| [#16](https://www.ncbi.nlm.nih.gov/pubmed) | Cellular Phone*[tiab] |
| [#15](https://www.ncbi.nlm.nih.gov/pubmed) | Cell Telephon*[tiab] |
| [#14](https://www.ncbi.nlm.nih.gov/pubmed) | Mobile Telephon*[tiab] |
| [#13](https://www.ncbi.nlm.nih.gov/pubmed) | Cell Phone*[tiab] |
| [#12](https://www.ncbi.nlm.nih.gov/pubmed) | Mobile Phone*[tiab] |
| [#11](https://www.ncbi.nlm.nih.gov/pubmed) | Cell Phone[Mesh] |
| [#10](https://www.ncbi.nlm.nih.gov/pubmed) | Mobile Authentic*[tiab] |
| [#9](https://www.ncbi.nlm.nih.gov/pubmed) | Medical App*[tiab] |
| [#8](https://www.ncbi.nlm.nih.gov/pubmed) | Mobile Based[tiab] |
| [#7](https://www.ncbi.nlm.nih.gov/pubmed) | Portable Software[tiab] |
| [#6](https://www.ncbi.nlm.nih.gov/pubmed) | Mobile Software[tiab] |
| [#5](https://www.ncbi.nlm.nih.gov/pubmed) | Software App*[tiab] |
| [#4](https://www.ncbi.nlm.nih.gov/pubmed) | Portable App*[tiab] |
| [#3](https://www.ncbi.nlm.nih.gov/pubmed) | Electronic App*[tiab] |
| [#2](https://www.ncbi.nlm.nih.gov/pubmed) | Mobile App*[tiab] |
| [#1](https://www.ncbi.nlm.nih.gov/pubmed) | Mobile Applications[Mesh] |

**EMBase (Elsevier)**

| No. | Query |
| --- | --- |
| #46 | #28 AND #45 |
| #45 | #29 OR #30 OR #31 OR #32 OR #33 OR #34 OR #35 OR #36 OR #37 OR #38 OR #39 OR #40 OR #41 OR #42 OR #43 OR #44 |
| #44 | pillsbot:ti,ab |
| #43 | pillidentif*:ti,ab |
| #42 | (pill NEAR/1 finder*):ti,ab |
| #41 | 'pill id':ti,ab |
| #40 | (pill NEAR/1 identif*):ti,ab |
| #39 | (medication NEAR/3 authentic*):ti,ab |
| #38 | (drug NEAR/3 authentic*):ti,ab |
| #37 | (pill NEAR/3 authentic*):ti,ab |
| #36 | substandard:ti,ab |
| #35 | fraudulent*:ti,ab |
| #34 | imitation*:ti,ab |
| #33 | adulterated:ti,ab |
| #32 | fake:ti,ab |
| #31 | falsified:ti,ab |
| #30 | counterfeit:ti,ab |
| #29 | 'counterfeit drug'/exp |
| #28 | #1 OR #2 OR #3 OR #4 OR #5 OR #6 OR #7 OR #8 OR #9 OR #10 OR #11 OR #12 OR #13 OR #14 OR #15 OR #16 OR #17 OR #18 OR #19 OR #20 OR #21 OR #22 OR #23 OR #24 OR #25 OR #26 OR #27 |
| #27 | ehealth:ti,ab |
| #26 | telehealth:ti,ab |
| #25 | mhealth:ti,ab |
| #24 | 'mobile health':ti,ab |
| #23 | iphone:ti,ab |
| #22 | ipad:ti,ab |
| #21 | 'handheld device':ti,ab |
| #20 | 'personal digital assistant'/exp |
| #19 | qr:ti,ab |
| #18 | smartphone*:ti,ab |
| #17 | (cellular NEAR/1 phone*):ti,ab |
| #16 | (cellular NEAR/1 telephone*):ti,ab |
| #15 | (cell NEAR/1 telephone*):ti,ab |
| #14 | (mobile NEAR/1 telephone*):ti,ab |
| #13 | (cell NEAR/1 phone*):ti,ab |
| #12 | (mobile NEAR/1 phone*):ti,ab |
| #11 | 'mobile phone'/exp |
| #10 | (mobile NEAR/1 authentic*):ti,ab |
| #9 | (medical NEAR/2 app):ti,ab |
| #8 | 'mobile based':ti,ab |
| #7 | (portable NEAR/2 software):ti,ab |
| #6 | (mobile NEAR/2 software):ti,ab |
| #5 | (software NEAR/1 app*):ti,ab |
| #4 | (portable NEAR/1 app*):ti,ab |
| #3 | (electronic NEAR/1 app*):ti,ab |
| #2 | (mobile NEAR/1 app*):ti,ab |
| #1 | 'mobile application'/exp |

**Cochrane Library (Wiley) 8-01-2020**

| #1 | MeSH descriptor: [Mobile Applications] explode all trees |
| --- | --- |
| #2 | (Mobile NEAR/1 App*):ti,ab,kw |
| #3 | (Electronic NEAR/1 App*):ti,ab,kw |
| #4 | (Portable NEAR/1 App*):ti,ab,kw |
| #5 | (Software NEAR/1 App*):ti,ab,kw |
| #6 | (Mobile NEAR/2 Software):ti,ab,kw |
| #7 | (Portable NEAR/2 Software):ti,ab,kw |
| #8 | (Mobile NEAR/1 Based):ti,ab,kw |
| #9 | (Medical NEAR/2 App*):ti,ab,kw |
| #10 | (Mobile NEAR/1 Authentic*):ti,ab,kw |
| #11 | MeSH descriptor: [Cell Phone] explode all trees |
| #12 | (Mobile NEAR/2 Phone*):ti,ab,kw |
| #13 | (Cell NEAR/2 Phone*):ti,ab,kw |
| #14 | (Mobile NEAR/1 Telephone*):ti,ab,kw |
| #15 | (Cell NEAR/1 Telephone*):ti,ab,kw |
| #16 | (Cellular NEAR/1 Phone*):ti,ab,kw |
| #17 | (Cellular NEAR/1 Telephone*):ti,ab,kw |
| #18 | Smartphone*:ti,ab,kw |
| #19 | QR:ti,ab,kw |
| #20 | MeSH descriptor: [Computers, Handheld] explode all trees |
| #21 | (Handheld NEAR/3 Device*):ti,ab,kw |
| #22 | iPad:ti,ab,kw |
| #23 | iPhone:ti,ab,kw |
| #24 | (Mobile NEAR/2 Health):ti,ab,kw |
| #25 | mHealth:ti,ab,kw |
| #26 | Telehealth:ti,ab,kw |
| #27 | eHealth:ti,ab,kw |
| #28 | #1 OR #2 OR #3 OR #4 OR #5 OR #6 OR #7 OR #8 OR #9 OR #10 OR #11 OR #12 OR #13 OR #14 OR #15 OR #16 OR #17 OR #18 OR #19 OR #20 OR #21 OR #22 OR #23 OR #24 OR #25 OR #26 OR #27 |
| #29 | MeSH descriptor: [Counterfeit Drugs] explode all trees |
| #30 | Counterfeit:ti,ab,kw |
| #31 | Falsified:ti,ab,kw |
| #32 | Fake:ti,ab,kw |
| #33 | Adulterated:ti,ab,kw |
| #34 | Imitation*:ti,ab,kw |
| #35 | Fraudulent*:ti,ab,kw |
| #36 | Substandard:ti,ab,kw |
| #37 | (Pill NEAR/1 Authentic*):ti,ab,kw |
| #38 | (Drug NEAR/1 Authentic*):ti,ab,kw |
| #39 | (Medication NEAR/1 Authentic*):ti,ab,kw |
| #40 | (Pill NEAR/1 Identif*):ti,ab,kw |
| #41 | (Pill NEAR/1 ID):ti,ab,kw |
| #42 | (Pill NEAR/1Finder*):ti,ab,kw |
| #43 | Pillidentif*:ti,ab,kw |
| #44 | PillsBot:ti,ab,kw |
| #45 | #29 OR #30 OR #31 OR #32 OR #33 OR #34 OR #35 OR #36 OR #37 OR #38 OR #39 OR #40 OR #41 OR #42 OR #43 OR #44 |
| #46 | #28 AND #45 |

**Global Health (OVID)**

| [# ▲](https://ovidsp-dc2-ovid-com.libproxy.tulane.edu/sp-4.03.0b/ovidweb.cgi?&S=ILNLFPEDBMEBKODCIPBKJEOGAHDAAA00&Sort+Sets=descending) | **Searches** |
| --- | --- |
| 1 | Mobile App*.ti,ab. |
| 2 | Electronic App*.ti,ab. |
| 3 | Portable App*.ti,ab. |
| 4 | Software App*.ti,ab. |
| 5 | Mobile Software.ti,ab. |
| 6 | Portable Software.ti,ab. |
| 7 | Mobile Based.ti,ab. |
| 8 | Medical App*.ti,ab. |
| 9 | Mobile Authentic*.ti,ab. |
| 10 | Mobile Phone*.ti,ab. |
| 11 | Cell Phone*.ti,ab. |
| 12 | Mobile Telephon*.ti,ab. |
| 13 | Cell Telephon*.ti,ab. |
| 14 | Cellular Phone*.ti,ab. |
| 15 | Cellular Telephon*.ti,ab. |
| 16 | Smartphone*.ti,ab. |
| 17 | QR.ti,ab. |
| 18 | Handheld Device.ti,ab. |
| 19 | iPad.ti,ab. |
| 20 | iPhone.ti,ab. |
| 21 | Mobile Health.ti,ab. |
| 22 | mHealth.ti,ab. |
| 23 | Telehealth.ti,ab. |
| 24 | eHealth.ti,ab. |
| 25 | or/1-24 |
| 26 | Counterfeit.ti,ab. |
| 27 | Falsified.ti,ab. |
| 28 | Fake.ti,ab. |
| 29 | Adulterated.ti,ab. |
| 30 | Imitation*.ti,ab. |
| 31 | Fraudulent*.ti,ab. |
| 32 | Substandard.ti,ab. |
| 33 | Pill Authentic*.ti,ab. |
| 34 | Drug Authentic*.ti,ab. |
| 35 | Medication Authentic*.ti,ab. |
| 36 | Pill Identif*.ti,ab. |
| 37 | Pill ID.ti,ab. |
| 38 | Pill Finder.ti,ab. |
| 39 | Pillidentif*.ti,ab. |
| 40 | PillsBot.ti,ab. |
| 41 | or/26-40 |
| 42 | 25 and 41 |

**Web Of Science**

| # 46 | #45 AND #28 |
| --- | --- |
| # 45 | #29 OR #30 OR #31 OR #32 OR #33 OR #34 OR #35 OR #36 OR #37 OR #38 OR #39 OR #40 OR #41 OR #42 OR #43 OR #44 |
| # 44 | TI=PillsBot |
| # 43 | TI=Pillidentif* |
| # 42 | TI=Pill Finder |
| # 41 | TI=Pill ID |
| # 40 | TI=Pill Identif* |
| # 39 | TI=Medication Authentic* |
| # 38 | TI=Drug Authentic* |
| # 37 | TI=Pill Authentic* |
| # 36 | TI=Substandard |
| # 35 | TI=Fraudulent* |
| # 34 | TI=Imitation* |
| # 33 | TI=Adulterated |
| # 32 | TI=Fake |
| # 31 | TI=Falsified |
| # 30 | TI=Counterfeit |
| # 29 | TS=Counterfeit Drugs |
| # 28 | #1 OR #2 OR #3 OR #4 OR #5 OR #6 OR #7 OR #8 OR #9 OR #10 OR #11 OR #12 OR #13 OR #14 OR #15 OR #16 OR #17 OR #18 OR #19 OR #20 OR #21 OR #22 OR #23 OR #24 OR #25 OR #26 OR #27 |
| # 27 | TI=eHealth |
| # 26 | TI=Telehealth |
| # 25 | TI=mHealth |
| # 24 | TI=Mobile Health |
| # 23 | TI=iPhone |
| # 22 | TI=iPad |
| # 21 | TI=Handheld Device |
| # 20 | TS=Computers, Handheld |
| # 19 | TI=QR |
| # 18 | TI=Smartphone* |
| # 17 | TI=Cellular Telephon* |
| # 16 | TI=Cellular Phone* |
| # 15 | TI=Cell Telephon* |
| # 14 | TI=Mobile Telephon* |
| # 13 | TI=Cell Phone* |
| # 12 | TI=Mobile Phone* |
| # 11 | TS=Cell Phone |
| # 10 | TI=Mobile Authentic* |
| # 9 | TI=Medical App* |
| # 8 | TI=Mobile Based |
| # 7 | TI=Portable Software |
| # 6 | TI=Mobile Software |
| # 5 | TI=Software App* |
| # 4 | TI=Portable App* |
| # 3 | TI=Electronic App* |
| # 2 | TI=Mobile App* |
| # 1 | TS=Mobile Applications |
