# Supplementary material for: Mobile apps for detecting falsified and substandard drugs: A systematic review
Source: PLoS One. 2021 Feb 4;16(2):e0246061. doi: 10.1371/journal.pone.0246061 (PMC7861418; doi:10.1371/journal.pone.0246061)
Supplement: Attachment — Submitted filename: Response to reviewers.docx [file pone.0246061.s004.docx]

1. Please ensure that your manuscript meets PLOS ONE's style requirements, including those for file naming. The PLOS ONE style templates can be found at

<https://journals.plos.org/plosone/s/file?id=wjVg/PLOSOne_formatting_sample_main_body.pdf> and

<https://journals.plos.org/plosone/s/file?id=ba62/PLOSOne_formatting_sample_title_authors_affiliations.pdf>

- **Done**

2. Please clarify the last date of your search for articles and applications.

- **Done: January 2020**

 3. You have provided a table of characteristics of the mobile applications; however, you have not summarized the characteristics (and quality) of the two studies you included.

**We found only two studies which evaluated the performance of four highly recognized apps (Epocrates, IBM Micromedex Drug Reference, Medscape and MedSnap).^[20,21]^ All these apps (the focus of our systematic review) are described in Table 1.**

**However, we expanded the narrative description of these studies and its quality as it can see below:**

*“Our search found a cross-sectional study that intended to compare Epocrates, Medscape, IBM Micromedex and Google for clinical management information designed for healthcare professionals for a one-week period.[20] Medical students at an academic hospital in the United States used a score for satisfaction of search and user interface based on a 1 to 5 scale, with 1 representing lowest quality and 5 describing the highest quality. The study concluded that Medscape (Satisfaction 4.92) was the most preferred free mobile app evaluated due to its interactive and educational features, followed by IBM Micromedex and Epocrates (Satisfaction 4.58 and 4.42, respectively). This study was used to complete MARS question #19 (evidence base) about whether the app had been trialed. Further, we found a preprint study whit the objective to evaluate the sensibility and specificity of MedSnap, that is not available on the app stores. In the study,[21]”*

“We used the NIH Study Quality Assessment Tools for the 2 included studies and in both cases a poor score was obtained, which means a high risk of bias. This demonstrates the lack of studies evaluating apps and the great need to carry out rigorous studies that evaluate the functioning and usefulness of apps for pill identification and eventually to detect SCMs. Likewise, the risk of bias assessment for RCTs could not be carried out since no study with this type of design was found.”

4. Neither publication bias nor study heterogeneity have been assessed. Please clarify why this is so.

**We performed a tabular and narrative synthesis of each identified mobile app. Since a meta-analysis is not applicable and there is no positive or negative result of apps’ descriptive reports,** **publication bias couldn’t be assessed. However, our exhaustive search and inclusion criteria prevent omissions of relevant studies.**

**We considered that a formal assessment of heterogeneity was not applicable. However, we describe and discuss the differences found among included apps.**

5. In your PRISMA checklist, you indicate that you did not present results of any risk assessment of bias across studies. However, in the Methods section, you indicate that risk of bias was assessed. Please present a summary of your findings if any.

**We summarized in Table 1 and 2 and Figure 2 the general characteristics of the reviewed apps and the app quality mean MARS scores available on platforms.**

**The MARS score is the instrument to assess the apps quality.**

**We added the quality assessment of the only two studies using NIH instrument for these cross-sectional studies.**

“We used the NIH Study Quality Assessment Tools for the 2 included studies and in both cases a poor score was obtained, which means a high risk of bias. This demonstrates the lack of studies evaluating apps and the great need to carry out rigorous studies that evaluate the functioning and usefulness of apps for pill identification and eventually to detect SCMs. Likewise, the risk of bias assessment for RCTs could not be carried out since no study with this type of design was found.”

6. Please include captions for your Supporting Information files at the end of your manuscript, and update any in-text citations to match accordingly. Please see our Supporting Information guidelines for more information: <http://journals.plos.org/plosone/s/supporting-information>.

- **Done**

Reviewers' comments:

Reviewer's Responses to Questions

**Comments to the Author**

1. Is the manuscript technically sound, and do the data support the conclusions?

The manuscript must describe a technically sound piece of scientific research with data that supports the conclusions. Experiments must have been conducted rigorously, with appropriate controls, replication, and sample sizes. The conclusions must be drawn appropriately based on the data presented.

Reviewer #1: Partly

2. Has the statistical analysis been performed appropriately and rigorously?

Reviewer #1: N/A

3. Have the authors made all data underlying the findings in their manuscript fully available?

The [PLOS Data policy](http://www.plosone.org/static/policies.action#sharing) requires authors to make all data underlying the findings described in their manuscript fully available without restriction, with rare exception (please refer to the Data Availability Statement in the manuscript PDF file). The data should be provided as part of the manuscript or its supporting information, or deposited to a public repository. For example, in addition to summary statistics, the data points behind means, medians and variance measures should be available. If there are restrictions on publicly sharing data—e.g. participant privacy or use of data from a third party—those must be specified.

Reviewer #1: Yes

4. Is the manuscript presented in an intelligible fashion and written in standard English?

PLOS ONE does not copyedit accepted manuscripts, so the language in submitted articles must be clear, correct, and unambiguous. Any typographical or grammatical errors should be corrected at revision, so please note any specific errors here.

Reviewer #1: Yes

**5. Review Comments to the Author**

Please use the space provided to explain your answers to the questions above. You may also include additional comments for the author, including concerns about dual publication, research ethics, or publication ethics. (Please upload your review as an attachment if it exceeds 20,000 characters)

**Reviewer #1**: My main concern with this paper is that **it is not a systematic review** of mobile apps for detecting falsified and substandard drugs (SF drugs). **Rather, it is a review** of 24 apps and 16 websites whose function is pill identification, and one app (MedSnap) whose function is to detect SF drugs.

**Considering the fulfillment of different definitions of systematic reviews, we considered that our study is indeed a systematic review:**

***DARE:** ≥ 4 criteria out of the first 5 (1-3 are mandatory)

**^#^Oxman and Guyatt 1991:** 1-4 & 6-7

**^#^Cochrane Collaboration, CRD, MOOSE, Potsdam Consultation, QUOROM, AHRQ:** 1-4 & 6-8

1. Were inclusion/exclusion criteria reported? Yes
2. Was the search adequate? Yes
3. Were the included studies synthesised? Yes, a Tabular and narrative synthesis
4. Was the validity of the included studies assessed?

*Yes, we used the NIH for the only 2 studies identified. Additionally, the other were descriptions of apps that were classified by MARS.*

5. Are sufficient details about the individual included studies presented? Yes, in tables and figures
6. Was the data extraction process adequate? Yes

*Data extraction and risk of bias (quality) assessment were also performed independently by these reviewers, with oversight from two senior reviewers (AC and AB).*

7. Was the study selection process adequate? Yes

*All unique articles were independently assessed by two reviewers (MD and TA) based on title and abstract. Those marked for inclusion, or whose title and abstract were not sufficient to determine inclusion, were then reviewed using the full text.*

8. Was ‘PICO’ used to focus the question(s)?

Not completely applicable, but PICO relevant components are derived from the presented objectives

*DARE. (Accessed 04/08/2011, 2011, at <http://www.crd.york.ac.uk/cms2web/AboutDare.asp>.)

\# Sander L, Kitcher H. Systematic and Other Reviews: Terms and Definitions Used by UK Organizations and Selected Databases. Systematic Review and Del-phi Survey. In: National Institute for Health and Clinical Excellence. London; 2006.

The authors used the MARS assessment tool to evaluate the usability of each app and website, but they didn’t actually assess any SF products. The authors point out that “we cannot extrapolate the identification of a pill by a device to the categorization of a product as counterfeit or substandard.” So, this manuscript reviewed the ease of use of “pill identification apps,” and that’s how it should be titled and pitched. I think the gaps in SF drug detection are serious and speak to a real problem in the world, but it’s not fair to evaluate apps designed for pill identification on this basis.

→change the title to remove the idea that the mss reviews mobile apps and websites whose function is to detect SF drugs

**Thank for such appropriate comment. We searched for direct assessments of SF products using these apps and this define the title and aims of our study. Unfortunately, we did not find any reported experiments of real-world SFs using these technologies. We highlighted this evidence gap and we recommended caution to interpret the pill identification quality scores due to the indirectness of the findings. A sentence was added in the result section to clarify this point.**

*‘’These reports referred to the usability and the ability of apps to correctly identify pills. However, did not find direct assessment of SF products in the real-world.”*

→add discussion of the pill identification task and its context

**We added this sentence in the discussion section**

*“In real life these apps are mostly used to identify pills by elderly people or people with difficulties to recognize medications but not to identify SCMs.”*

→add discussion of the technical challenges for detecting SF drugs via pill image analysis (this is why none of the apps/sites that rely on the user inputting pill color/shape/imprint data are capable of SF detection)

**While detecting SF drugs via pill image analysis does not allow proper SC drugs detection it might help as an initial screening test, particularly with negative results.**

Most of the pill identification apps and websites, such as the highly rated Medscape website, ask the user to input pill imprint numbers, color, and shape, and match that information to a market-specific database. Only the most carelessly made fake products would fail to match at this level. The authors say (line 197) that “developers claim that the apps have the capacity to also detect SCMs”.
→I would like this statement to be backed up by data about which pill identifier products make this claim.

**Two apps claim to have the capacity to detect SCMs: Medsnap and CheckFake**

Tools such as MedSnap utilize pill photographs which can be compared to a library of known “good” pills via image analysis tools. This method detects subtle differences between the imprint or tablet size/shape/color of a genuine manufacturer and a counterfeit version. (That was the study they did in Laos on fake artesunate and coartem.)
→There is no evidence that this kind of image analysis can detect substandard products.

**MedSnap detects SF drugs via pill image analysis. Although it does not allow a proper SC drugs detection, it still might help as an initial screening test, that could be particularly useful if it discard SC drugs.**

→The authors might consider discussing the CD3/CDx device, which is another image analysis tool that uses multispectral imaging of pharmaceuticals—there is a published field testing record for a range of SF products.

Ranieri N et al (2014) Evaluation of a new handheld instrument for the detection of counterfeit artesunate by visual fluorescence comparison. Am J Trop Med Hyg 91:920–924. <https://doi.org/10.4269/ajtmh.13-0644>

**Thank you but our systematic review was focused only on mobile apps.**
