# Supplementary material for: Mobile apps for detecting falsified and substandard drugs: A systematic review
Source: PLoS One. 2021 Feb 4;16(2):e0246061. doi: 10.1371/journal.pone.0246061 (PMC7861418; doi:10.1371/journal.pone.0246061)
Supplement: Attachment — Submitted filename: Responses Reviewers comments 2.docx [file pone.0246061.s005.docx]

17/12/2020

PONE-D-20-22666R1

**Mobile apps for detecting falsified and substandard drugs: A systematic review**

PLOS ONE

**Comments to the Author**

1. **If the authors have adequately addressed your comments raised in a previous round** of review and you feel that this manuscript is now acceptable for publication, you may indicate that here to bypass the “Comments to the Author” section, enter your conflict of interest statement in the “Confidential to Editor” section, and submit your "Accept" recommendation.

Reviewer #1: (No Response)

**2. Is the manuscript technically sound, and do the data support the conclusions?**

The manuscript must describe a technically sound piece of scientific research with data that supports the conclusions. Experiments must have been conducted rigorously, with appropriate controls, replication, and sample sizes. The conclusions must be drawn appropriately based on the data presented.

Reviewer #1: Yes

**3. Has the statistical analysis been performed appropriately and rigorously?**

Reviewer #1: N/A

**4. Have the authors made all data underlying the findings in their manuscript fully available?**

The PLOS Data policy requires authors to make all data underlying the findings described in their manuscript fully available without restriction, with rare exception (please refer to the Data Availability Statement in the manuscript PDF file). The data should be provided as part of the manuscript or its supporting information, or deposited to a public repository. For example, in addition to summary statistics, the data points behind means, medians and variance measures should be available. If there are restrictions on publicly sharing data—e.g. participant privacy or use of data from a third party—those must be specified.

Reviewer #1: Yes

**5. Is the manuscript presented in an intelligible fashion and written in standard English?**

PLOS ONE does not copyedit accepted manuscripts, so the language in submitted articles must be clear, correct, and unambiguous. Any typographical or grammatical errors should be corrected at revision, so please note any specific errors here.

Reviewer #1: Yes

**6. Review Comments to the Author**

Please use the space provided to explain your answers to the questions above. You may also include additional comments for the author, including concerns about dual publication, research ethics, or publication ethics. (Please upload your review as an attachment if it exceeds 20,000 characters)

Reviewer #1: PONE-D-20-22666R1 Mobile apps for detecting falsified and substandard drugs: A systematic review

I am flabbergasted by the general lack of documentation and regulatory oversight of these products and think it’s worthwhile for the mss to be published to highlight this evidence gap.

Thank you for the comment, we have reflected now this key message in the conclusions of the abstract

*“In conclusion, we found a remarkable evidence gap about the accuracy of mobile apps in detecting SCMs.”*

The conclusions of the manuscript already clearly reflected this concept

*“Our systematic review highlighted an important evidence gap in diagnostic accuracy of mobile apps detecting SCMs, and there is a need for primary studies addressing this issue.”*

I’m still having a problem with the title. The authors conducted a systematic review of apps and websites that are used for pill identification. Only two of them make any claims to detect falsified or substandard products, and only one of those has any evidence base to support the claims. Can you have a systematic review of one manuscript?-->Maybe, call it “a systematic review of pill identification apps with potential utility for detecting SF drugs”?

Dear reviewer, our research question was about mobile apps for detecting falsified and substandard drugs and our protocol was registered in PROSPERO (CRD42020163075) under this name. We consider a good research practice to be consistent with the protocol, regardless of the findings finally obtained.

The name of a systematic review refers to the research question that guide the search strategy of a systematic review. Is not only possible that a systematic review has only one included study, but may be a dessert systematic review with cero included studies (There are multiple examples in the Cochrane Library). Of course, as you suggested, we have reinforced the first key message highlighting the identified evidence gap.

Additionally, the pill identification functionality could indirectly help to detect falsified and substandard drugs.

This limitation in the identified evidence is also clearly discussed in the manuscript.

-->Please add the references to the MedSnap and CheckFake distributor’s claims of SF detection to the mss.

Done. These are the references included in the manuscript now:

22.	Medsnap Verify Services. 2020 [cited 17 Dec 2020]. Available: <https://www.medsnap.com/verify/>

23.	Google play. CheckFake (dlt.sg App for drug counterfeit). 2020 [cited 17 Dec 2020]. Available: <https://play.google.com/store/apps/details?id=sg.dlt.checkfake&hl=es_419>

From the app store entry, CheckFake is a bar code checker, not a pill checker, and does not seem to be supported by or connected with any company—I didn't see a website or published literature on it. Pill checkers need to be maintained and upgraded as new brands or drugs enter the market, or as the app is used in markets that offer different brands. In the future it would be interesting to evaluate whether each app is "really" available in different regions, eg, whether there is a company behind it to support users and upgrade the app and whether the product really works for the brands found in Europe, America, Africa, etc.

Thank you for the comment. We have corrected the information about the CheckFake app. We had to include it because this reference met our inclusion criteria, however our assessment of the quality and reliability of the information of this app was evaluated with the worst of the values of the MARS questionnaire.

7. PLOS authors have the option to publish the peer review history of their article (what does this mean?). If published, this will include your full peer review and any attached files.

If you choose “no”, your identity will remain anonymous but your review may still be made public.

Do you want your identity to be public for this peer review? For information about this choice, including consent withdrawal, please see our Privacy Policy.

Reviewer #1: Yes: Marya Lieberman
